# Prenatal Alcohol Exposure Results in Sex-Specific Alterations in Circular RNA Expression in the Developing Mouse Brain

**DOI:** 10.3389/fnins.2020.581895

**Published:** 2020-11-09

**Authors:** Praveen Paudel, Caroline Pierotti, Evelyn Lozano, Stephen K. Amoah, Amy S. Gardiner, Kevin K. Caldwell, Andrea M. Allan, Nikolaos Mellios

**Affiliations:** ^1^Department of Neurosciences, University of New Mexico School of Medicine, Albuquerque, NM, United States; ^2^Department of Biology, University of New Mexico, Albuquerque, NM, United States; ^3^Autophagy Inflammation and Metabolism (AIM) Center, Albuquerque, NM, United States; ^4^Department of Cell Biology and Physiology, University of New Mexico School of Medicine, Albuquerque, NM, United States

**Keywords:** circular RNA, circular RNAs, fetal alcohol, prenatal alcohol exposure, sexual dimorphism

## Abstract

Fetal alcohol spectrum disorders (FASD) are heterogeneous disorders associated with alcohol exposure to the developing fetus that are characterized by a range of adverse neurodevelopmental deficits. Despite the numerous genomics and genetic studies on FASD models, the comprehensive molecular understanding of the mechanisms that underlie FASD-related neurodevelopmental deficits remains elusive. Circular RNAs (circRNAs) are a subtype of long non-coding RNAs that are derived from back-splicing and covalent joining of exons and/or introns of protein-coding genes. Recent studies have shown that circRNAs are highly enriched in the brain, where they are developmentally regulated. However, the role of the majority of brain-enriched circRNAs in normal and pathological brain development and function has not been explored yet. Here we carried out the first systematic profiling of circRNA expression in response to prenatal alcohol exposure (PAE) in male and female embryonic day 18 (E18) whole brains. We observed that the changes in circRNA expression in response to PAE were notably sex-specific and that PAE tended to erase most of the sex-specificity in circRNA expression present in control (saccharin-treated) mice. On the other hand, RNA sequencing (RNA-seq) in the same samples showed that changes in protein-coding gene expression were not predominantly sex-specific. Using circRNA quantitative real-time PCR (qRT-PCR), we validated that *circSatb2*, which is generated from the special AT-rich sequence-binding protein 2 (*Satb2*) gene, is significantly upregulated in the brain of E18 male PAE mice. We also show that *circPtchd2*, a circRNA synthesized from dispatched RND transporter family member 3 (*Disp3*, also known as *Ptchd2*), exhibits significantly higher expression in E18 control but not PAE female mouse brain relative to males. Taken together, our results demonstrate that PAE differentially alters circRNA expression in the developing brain in a sex-specific manner.

## Introduction

Fetal alcohol spectrum disorder (FASD) encompasses a range of heterogeneous developmental disorders with nervous system abnormalities including learning and memory deficits (Brady et al., 2012; Carter et al., 2016), alterations in behavioral flexibility (Marquardt et al., 2020), and reduced brain volume and cortical thickness (Treit et al., 2017). It is estimated that at least 10% of women consume alcohol when pregnant (Riley et al., 2011) and that between 2 and 5% of school-aged children suffer from some form of FASD, with a subset of them exhibiting severe Fetal Alcohol Syndrome abnormalities (May et al., 2009). FASD, which is characterized by deficits in cognitive and executive functions, could manifest as a plethora of neurodevelopmental abnormalities and long-lasting neuropsychiatric impairments, whose severity depends on the dose, timing, and duration of prenatal alcohol exposure (PAE) (Sokol et al., 2003; Chudley et al., 2005; Valenzuela et al., 2012).

Despite the progress that has been made in identifying protein-coding genes that are altered in the brain as a result of prenatal alcohol (EtOH) exposure and could influence brain development and function, the detailed molecular networks that underlie FASD-mediated neurodevelopmental disturbances are not fully understood. Previous studies have reported aberrant expression of the evolutionarily-conserved small non-coding RNAs (ncRNAs) known as microRNAs (miRNAs) in mouse models of FASD (Balarama et al., 2012; Pappalardo-Carter et al., 2013; Tapocik et al., 2014; Darcq et al., 2015; Mahnke et al., 2018). Furthermore, long ncRNAs (lncRNAs) are abundantly expressed in the human brain and are involved in the control of diverse transcriptional and posttranscriptional processes (Mercer et al., 2009; Qureshi and Mehler, 2012; Mercer and Mattick, 2013). Recently, a novel subset of lncRNAs known as circular RNAs (circRNAs), which are predominately derived from the covalent joining of back-spliced exons, were found to be particularly enriched in the mammalian brain and abundantly expressed in synapses (Hansen et al., 2013; Memczak et al., 2013; Guo et al., 2014; Rybak-Wolf et al., 2014; You et al., 2015). CircRNAs are generated from exons and introns of protein-coding genes and display neuronal activity- and developmental stage-dependent expression profiles (Rybak-Wolf et al., 2014; Gruner et al., 2016; Reddy et al., 2017; Zimmerman et al., 2020). Their biogenesis has been shown to be promoted by interactions between complementary intronic repeat sequences and the binding of RNA-binding proteins (RBPs) close to the circRNA splice junction, but inhibited by factors that promote linear splicing and proteins that can disrupt the interactions between the intronic complementary regions (Li et al., 2018; Kristensen et al., 2019). The use of advanced annotation tools and deep sequencing of ribosomal RNA (rRNA)-depleted and RNase R-digested RNA samples has revealed the existence of tens of thousands of circRNAs (Hansen et al., 2013; Memczak et al., 2013; Guo et al., 2014; Rybak-Wolf et al., 2014; You et al., 2015). Interestingly, circRNA expression has been shown to be altered in various psychiatric disorders (Zimmerman et al., 2020), autism (Chen et al., 2020), cocaine addiction (Bu et al., 2019), and Alzheimer’s disease (Dube et al., 2019). Among the potential functions of circRNAs are interactions with RBPs, sequestration of miRNAs, and transcriptional control of gene expression (Hentze and Preiss, 2013; Barrett and Salzman, 2016; Gokool et al., 2020). Interestingly, manipulating circRNA expression in the mouse brain can result in altered synaptic and activity-dependent gene expression, as well as in changes in neuronal function and behavior in adult mice (Piwecka et al., 2017; Zimmerman et al., 2020). Furthermore, a circRNA can be essential for maintaining neural cells in a progenitor state (Suenkel et al., 2020), enriched in the neuropil, and increased during neuronal maturation (Rybak-Wolf et al., 2014; You et al., 2015), suggesting that circRNAs could also have an important role during embryonic and fetal brain development. Despite the above, very little is known about the role of circRNAs in neurodevelopmental disorders, including FASD.

Using circRNA array profiling, we systematically profiled the expression of circRNAs in the brain of an FASD mouse model of PAE, previously shown to result in FASD-like neurodevelopmental alterations (Brady et al., 2012; Allan et al., 2014; Marquardt et al., 2020). We observed that PAE not only affected circRNA levels in the prenatal brain in a sex-specific manner, but also normalized circRNA expression changes observed between control male and female brains. Using RNA sequencing (RNA-seq) specialized for linear mRNA detection, we observed that the sex-specificity in PAE-mediated alterations in circRNAs was not accompanied by similar changes in protein-coding gene expression. Furthermore, we validated the sex-specific expression or alterations following PAE of two circRNAs stemming from genes known to be important for prenatal brain development, *circPtchd2*, and *circSatb2*, using circRNA-specific quantitative real-time PCR (qRT-PCR). Our data suggest that *circSatb2*, which is derived from the special AT-rich sequence-binding protein 2 (*Satb2*) gene, is significantly upregulated in the brain of E18 male but not female PAE mice. Furthermore, *circPtchd2*, a circRNA derived from dispatched RND transporter family member 3 (*Disp3*, also known as *Ptchd2*), was found to have higher expression in control (saccharin-treated) female vs. male E18 brains. Taken together, our data suggest that prenatal brain circRNA but not protein-coding gene expression is regulated in a sex-specific manner by PAE.

## Materials and Methods

### Animals

All procedures were approved by the University of New Mexico Health Sciences Center Institutional Animal Care and Use Committee. In all studies, brains were collected from fetuses of dams euthanized by decapitation without anesthesia.

### Prenatal Exposure

**FIGURE 1:**
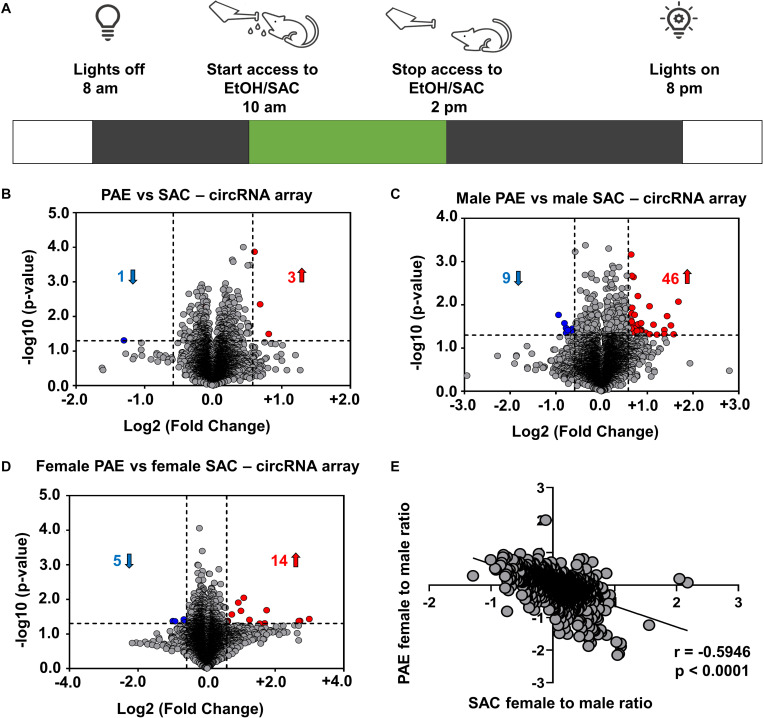
Changes in circRNA expression in embryonic brain following PAE display sex dimorphism. **(A)** Schematic representation of “drinking in the dark” PAE model of FASD. As described above, female mice were subjected to voluntary drinking of either 0.066% (w/v) saccharin sweetened solution (control) or 10% (w/v) ethanol in 0.066% (w/v) saccharin sweetened solution for 4 h/day during mating and throughout the gestational period. **(B)** Volcano plot showing overall fold changes in the 10,000 most abundant circRNAs in PAE (*N* = 6 with half being male and half female) compared to SAC (control) E18 whole brain (*N* = 6 with half being male and half female). Overall, only 3 circRNAs were upregulated and 1 circRNA was downregulated (1.5-fold changes, *p* < 0.05). **(C)** Volcano plot showing fold changes in circRNAs expression in male PAE compared to male SAC E18 whole brain. 46 circRNAs were upregulated and 9 circRNAs were downregulated with 1.5-fold changes (*p* < 0.05). **(D)** Volcano plot showing fold changes in circRNAs expression in female PAE compared to female SAC E18 brain. 14 circRNAs were upregulated and 5 circRNAs were downregulated with 1.5-fold changes (*p* < 0.05). In all volcano plots **(B–D)**, X-axis represents log2 fold change (dotted line = 1.5-fold), Y-axis represents –log10 *p*-value (dotted line: *p* = 0.05). **(E)** Spearman correlation of female to male ratio for each circRNA in either control SAC (female SAC vs. male SAC) or PAE (female PAE vs. male PAE). A robust negative correlation (Spearman correlation coefficient *r* = –0.5946, two-tailed *p*-value *p* < 0.0001) was observed between female to male ratios in SAC and PAE mice.

WT C57BL/6J mice derived from Jackson Laboratory were maintained on a reverse 12 h dark/light schedule (lights on at 8:00 p.m.) in single-housed cages. We utilized a well-validated PAE paradigm (Brady et al., 2012) that involves giving adult female mice access to a solution of either 10% (w/v) ethanol and 0.066% (w/v) saccharin or 0.066% (w/v) saccharin (control) for 4 h per day (Figure 1A). This model produced blood EtOH levels of 80–90 mg/dL of plasma (Brady et al., 2012, 2013). After validating consistent drinking, mice were given access to these solutions during mating and for the duration of their pregnancy. They were euthanized at the end of their pregnancy and whole brain samples were extracted from embryonic day 18 (E18) male and female pups and stored in a −80°C freezer.

### Quantification of CircRNA and mRNA Expression

Total RNA was isolated using the miRNeasy RNA isolation kit (Qiagen, Hilden, Germany). Following RNA extraction, RNA concentration and quality was assayed through NanoDrop 2000 spectrophotometer and Qubit 3 (Thermo Fisher Scientific, Waltham, MA). Reverse transcription was performed using the SuperScript IV First-Strand Synthesis System (Thermo Fisher Scientific) with random hexamers for circRNA and oligo-dT primers for linear mRNA RNA detection. cDNA was then used together with custom made, validated, and sequence-verified circRNA and mRNA primers or TaqMan mRNA primers (Thermo Fisher Scientific) for mRNA qRT-PCR. *18S rRNA* was used as a normalizer for mRNA detection, whereas *circTulp4* or *18S rRNA* was used for circRNA normalization. For qRT-PCR quantification the following formula was used: Relative value = 2^Ctnormalizer/2^CtmRNA or circRNA.

### CircRNA Profiling in Mouse Brain

CircRNA expression in E18 whole brain was profiled using Arraystar Mouse Circular RNA Microarray service (Arraystar Inc., Rockville, MD). The circRNA array platform consisted of 14,236 probes designed by the manufacturer to detect the unique circRNA splice junction utilizing several circRNA sources (Memczak et al., 2013; Guo et al., 2014; You et al., 2015). Briefly, 800 ng of extracted total RNA (see above) was treated with RnaseR (3 h at 37°C of ribonuclease R, 20 U/μL, Epicenter, Madison WI) to digest linear RNA and enrich circRNA. Random primers were then used to amplify and transcribe the enriched circRNAs into fluorescent cRNAs per Arraystar Super RNA Labelling protocol (Arraystar Inc.). Using Arraystar Mouse Circular RNA arrays (8 × 15K, Arraystar, Inc.), the labeled cRNAs were hybridized and incubated in an Agilent hybridization oven for 17 h at 65°C (Agilent Technologies, Santa Clara, CA). The slides were washed and eventually scanned using the Agilent Scanner G2505C (Agilent Technologies). All circRNA profiling data have been deposited in the Mendeley online data repository^1^.

### RNA Sequencing in E18 Whole Brain Samples

We used Arraystar Inc. service for library preparation and RNA sequencing (RNA-seq). Briefly, 1 μg of Total RNA was used for library construction utilizing Kapa Stranded RNA-seq library kit (Illumina). Illumina HiSeq 4,000 with a read length of 150 bps was used for paired end RNA-seq, and image analysis and base calling were performed using solexa platform v1.8. FastQC software was used to measure sequence quality and then, cut adapt software for adapter trimming and filtering. Using HiSat 2 software, reads were then aligned to mouse genome GRCm28 and StringTie was used for estimating transcript abundance for each sample. Eventually, R package Ballgown was utilized for calculating FPKM value for gene and transcript levels. Data related to this RNA-seq have been submitted to GEO (approval number = GSE154018).

### Statistical Analysis

Statistical analysis was performed using the GraphPad Prism analysis software. Normal distribution of data was evaluated via Shapiro–Wilk normality test. For comparisons between two groups a two-tailed Student’s *t*-test or Mann-Whitney test was used. Comparisons between more than two groups were performed via one-way Analysis of Variance (ANOVA) with Sidak’s multiple comparisons correction. The Robust regression and outlier removal (ROUT) method was utilized to identify significant outliers (*Q* = 1%). Pearson’s or Spearman’s correlation coefficients and two-tailed *p*-values were used for calculating correlations. All qPT-PCR experiments were performed blind to each sample’s identity.

## Results

### Changes in CircRNA but Not mRNA Expression in Embryonic Brain Following PAE Display Sex Dimorphism

In order to examine the effects of *in utero* alcohol exposure on circRNA expression during prenatal development, we utilized a well-validated PAE mouse model (Figure 1A) and employed circRNA array technology that analyzes up to 14,236 circRNAs in RNaseR-treated total RNA from 12 whole brains of E18 male and female mice, whose mothers consumed ethanol (PAE mice; *N* = 6 with half being male and half female) or saccharin control (SAC mice; *N* = 6 with half being male and female) during the whole duration of their pregnancy. Looking at the top 10,000 most highly abundant in E18 brain and differentially expressed circRNAs in PAE vs. SAC mice regardless of sex, we noticed that there was only a small subset of circRNAs that were significantly altered (Figure 1B; only 3 circRNAs were upregulated and 1 circRNA was downregulated with a cutoff of 1.5-fold change and *p* < 0.05). On the other hand, when circRNA expression was compared in a sex-specific manner, a much larger subset of circRNAs were found to be significantly altered (Figures 1C,D and Supplementary Table 1). Specifically, in male mice, 46 circRNAs were significantly upregulated and 9 circRNAs were downregulated in PAE compared to SAC E18 whole brain based on the same cutoff criteria (1.5-fold changes, *p* < 0.05; Figure 1C). In addition 14 circRNAs were upregulated and 5 circRNAs were downregulated and (1.5-fold changes, *p* < 0.05) in female PAE compared to female SAC mice (Figure 1D), suggesting that PAE alters circRNA expression in the prenatal brain in a sex-specific manner. Of note, none of the male PAE-altered circRNAs overlapped with the female PAE-altered circRNAs (Supplementary Table 1). We then calculated the female to male ratio for each circRNA in either control SAC (female SAC vs. male SAC) or PAE (female PAE vs. male PAE) mice and compared these ratios for each circRNA. Intriguingly, we observed a robust negative correlation (*r* = −0.5946, *p* < 0.0001; Figure 1E) between female to male ratios in SAC and PAE mice. This suggests that in addition to the sex-specific alterations in circRNA expression, PAE also preferentially erases or reverses most of the sex-specific changes in circRNA expression that physiologically occur in the developing brain.

**FIGURE 2:**
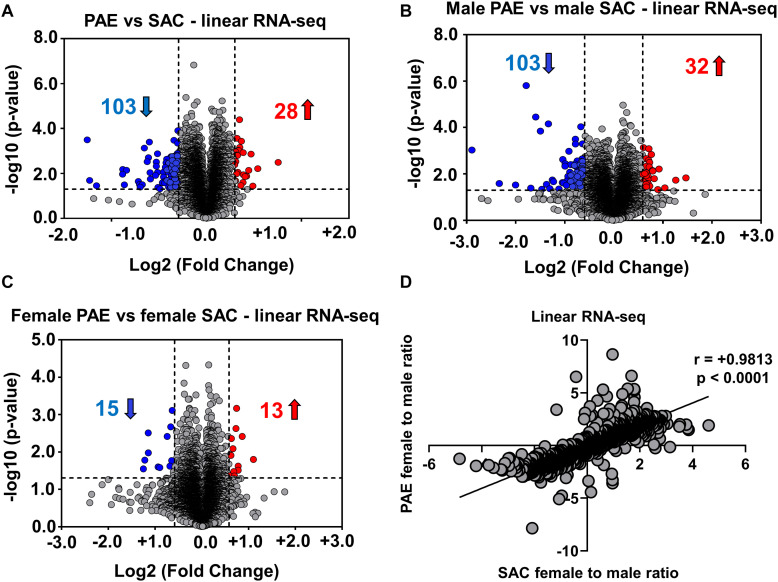
Alterations in linear mRNA expression in embryonic brain following PAE. **(A)** Volcano plot showing overall fold changes in linear mRNAs in the same (Figure 1B) E18 whole brain samples. When expression in PAE was compared to SAC E18 whole brain, overall 28 linear mRNAs were upregulated and 103 linear mRNAs were downregulated (1.5-fold changes, *p* < 0.05). **(B)** Volcano plot showing a similar number of linear mRNAs being significantly altered when compared between male PAE and male SAC groups. 32 linear RNAs were upregulated and 103 linear RNAs were downregulated (1.5-fold changes, *p* < 0.05). **(C)** Volcano plot showing fewer linear mRNAs to be significantly altered when compared between female PAE and female SAC E18 whole brain. 13 linear mRNAs were upregulated and 15 linear mRNAs were downregulated with 1.5-fold changes (*p* < 0.05). In all volcano plots **(A–C)**, *X*-axis represents log2 fold change (dotted line = 1.5-fold), *Y*-axis represents –log10 *p*-value (dotted line: *p* = 0.05). **(D)** A robust positive correlation (Spearman correlation coefficient *r* = 0.9813, two-tailed *p*-value *p* < 0.0001) between female to male ratios in SAC and PAE mice was observed in linear mRNAs.

In order to determine if such an effect is limited to circRNA expression, we then performed RNA sequencing following poly-A selection, so as to only detect mRNA expression in the same 12 E18 whole brain samples from PAE and SAC mice. In contrast to our circRNA profiling findings, our results indicated that 103 linear RNAs were downregulated and 28 linear RNAs were upregulated (1.5-fold changes, *p* < 0.05) in comparison to SAC groups regardless of sex (Figure 2A). Looking at changes in mRNA levels between male PAE vs. Control E18 whole brains, we observed a similar number of downregulated and upregulated mRNAs (103 increases and 32 decreased; Figure 2B). On the other hand, few mRNAs were found to be altered in female PAE vs. female SAC whole brains (15 downregulated, 13 upregulated; Figure 2C). We then calculated the female to male ratio for each mRNA in control SAC (female SAC vs. male SAC) or PAE (female PAE vs. male PAE) samples and compared these ratios again for each mRNA. We then inquired whether some of the significantly altered circRNAs in either male or female PAE could be exhibiting significant correlations with mRNA levels of their parent genes. We found only 9 significantly altered circRNAs with positive correlations to mRNA levels and 5 with negative correlations (two-tailed *p*-value < 0.1 and Pearson’s coefficient of > 0.5 or < −0.5). Furthermore, we found that unlike what was seen in circRNA expression, there was a robust positive correlation between female to male mRNA expression ratios in SAC and PAE mice (Figure 2D) via RNA-seq, suggesting that sex dimorphism in mRNA expression was predominately preserved despite PAE. This suggests that although PAE changes overall prenatal brain mRNA expression, it does so in a sex-independent manner.

### Differentially Expressed in Prenatal Brain CircRNAs in Male and Female PAE Mice Are Preferentially Generated From Genes With Relevance to Neuronal Development and Function

**FIGURE 3:**
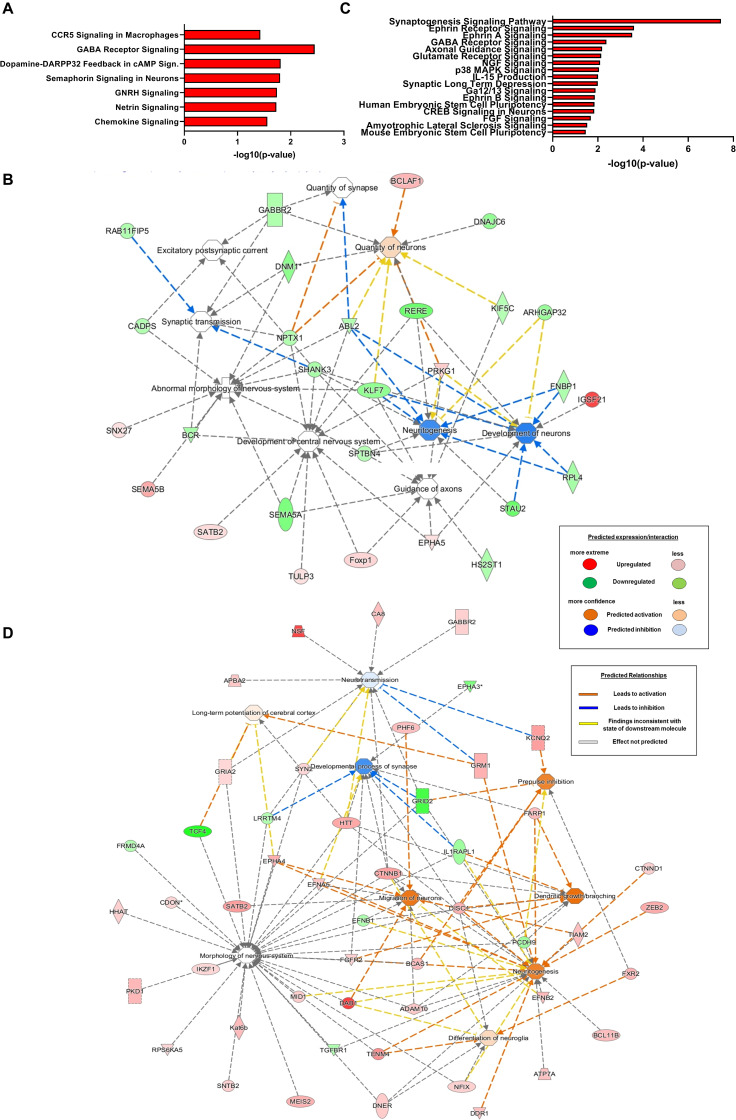
Differentially expressed circRNAs in the prenatal brain following PAE are preferentially generated from genes with relevance to neuronal development and function. **(A–D)** Molecular network and pathway analysis of host genes of PAE-altered *circRNAs* in a sex-specific manner (Ingenuity Pathway Analysis). **(A,B)** Canonical pathways of host genes of differentially expressed circRNAs in female PAE vs. female SAC E18 whole brain (**A**; cutoff lowered to 1.25-fold with *p* < 0.05) and male PAE vs. male SAC E18 whole brains (**B**; cutoff lowered to 1.25-fold with *p* < 0.05). **(C**,**D)** Molecular networks of host genes of differentially expressed circRNAs in female PAE vs. female SAC E18 whole brain **(C)** male PAE vs. male SAC E18 whole brains. **(D)** The same cutoff criteria were used as in **(A,B)**. A table explaining the depiction of predicted expression, interactions, and relationships between these networks is also displayed.

In order to determine the pathways and molecular networks that could be formed from the genes that generate differentially expressed circRNAs in the developing brain of male and female PAE mice, we used Ingenuity Pathway analysis of differentially altered circRNAs in either female PAE vs. female SAC or male PAE vs. male SAC E18 whole brains (cutoff lowered to 1.25-fold with *p* < 0.05). Interestingly, our results in female mice suggested that GABA receptor signaling, semaphoring signaling in neurons, and dopamine-DARPP32 feedback in cAMP signaling were among the top predicted pathways formed by the genes the harbor differentially expressed circRNAs due to PAE (Figure 3A). Looking at the molecular networks that could be formed by these genes, we identified many relevant to FASD networks harboring numerous genes generating PAE-altered circRNAs in female mice, such as development or abnormal morphology of the nervous system, development of neurons and neuritogenesis, and quantity of neurons and axonal guidance (Figure 3B). Smaller in size networks were also associated with synaptogenesis and synaptic transmission (Figure 3B), a process that starts around E15 but picks up mostly after postnatal brain development. Looking at genes that generate circRNAs preferentially altered in male PAE E18 brain, we noticed that synaptogenesis was the top canonical pathway, followed by Ephrin receptor and Ephrin A signaling, GABA receptor signaling, axonal guidance, glutamate receptor and CREB signaling, and synaptic long-term depression (Figure 3C). Focusing on the molecular networks that are formed from these genes, we noticed that in contrast to what was seen if female mice, there was a similar size for networks related to neuronal and neurite development, neuronal differentiation and migration, and synaptogenesis and synaptic plasticity (Figure 3D). Notably, genes that harbored differentially expressed circRNAs in male PAE brain were also associated with neurotransmission, long term potentiation, and prepulse inhibition (Figure 3D). These results taken together suggest, that circRNAs altered in E18 brain of male mice following PAE are more likely to be derived from genes associated with later stages of neuronal development and function.

### Sex-Specific Changes in *CircPtchd2* and *CircSatb2* Expression in Prenatal Brain

**FIGURE 4:**
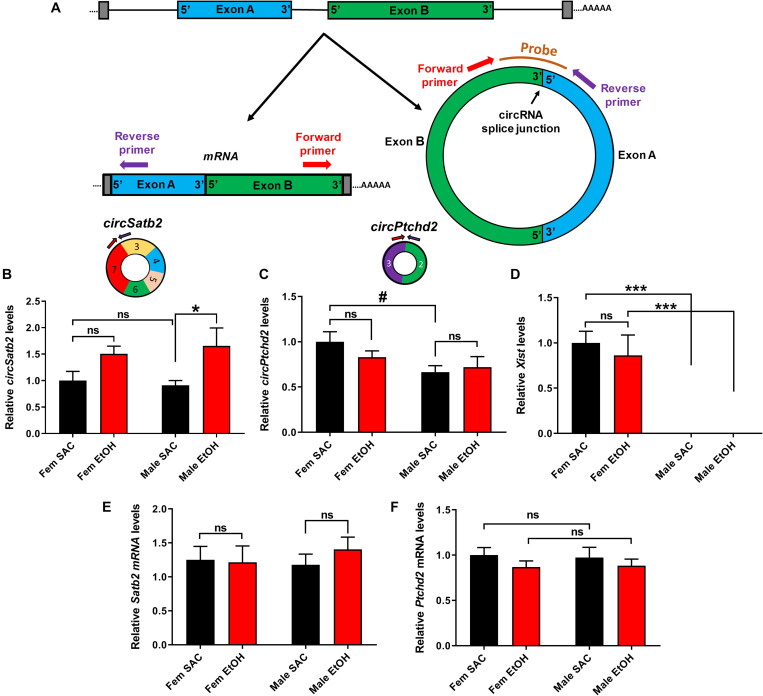
Validation of sex-dimorphic circRNA expression via circRNA-specific qRT-PCR. **(A)** Schematic representation of a simplified example of circRNA biogenesis resulting from back-splicing events of exon A and Exon B from a hypothetical precursor mRNA. The divergent primers for circRNA qRT-PCR validation were designed such that only the back-spliced and not linear junction were targeted as shown in the figure. **(B,C)** Bar graph showing mean + SEM relative to FEM SAC Control *circSatb2* **(B)** and *circPtchd2* **(C)** expression. Circles depict the schematic representation of *circPtchd2* and *circSatb2* formation from their respective exons. **(D)** Bar graph showing mean ± SEM levels of the female-specific lncRNA *Xist* expression (positive control). **(E,F)** No significant difference in linear *Satb2* and *Ptchd2* mRNA expression. In **(B–F)** ^#^0.05 < *p* < 0.10, *p < 0.05, ***p < 0.001 based on one-way Analysis of Variance (ANOVA) with Sidak’s multiple comparisons correction. In all figures, circRNA and mRNA quantification was done via qRT-PCR (normalized to *circTulp4* for circRNAs or *18S rRNA* for mRNAs). Bar graphs showing mean ± SEM relative to female SAC (control). Normalized values were divided to the mean of each control group and the relative to control ratios were graphed as means ± SEM. *N* = 4–6 per group.

In order to further explore the effects of PAE in sex-dependent circRNA expression, we used circRNA-specific qRT-PCR with divergent primers spanning the unique circRNA backsplicing region (Figure 4A). For further validation, we first chose *circSatb2*, a circRNA that was significantly increased in male PAE vs. male SAC brain (Supplementary Table 1), which is derived from special AT-rich sequence-binding protein 2 (*Satb2*), a gene known to regulate brain development and implicated in FASD (Alcamo et al., 2008; Britanova et al., 2008; Rosenfeld et al., 2009; Mandal et al., 2015). In addition, we chose to validate changes in *circPtchd2*, a circRNA derived from dispatched RND transporter family member 3 (*Disp3*, also known as *Ptchd2*), which displayed sex-specific changes in control SAC E18 brains, but also appeared to significantly increase in male PAE vs. male SAC brains (Supplementary Table 1). *CircPtchd2* is generated from the backsplicing events of exons 2–3 of *Ptchd2* and *circSatb2* is derived from exons 3–7 of the *Sabt2* gene. Our circRNA-specific qRT-PCR data validated that *circSatb2* is significantly increased in male PAE vs. male SAC E18 brains, with no changes in female PAE vs. SAC E18 brains (Figures 4A,B). Of note, an additional circRNA derived from *Satb2*, with lesser overall expression was identified via circRNA profiling to be also increased in male PAE brains (Supplementary Table 1), but we were unable to detect it via qRT-PCR (data not shown). Similar to our circRNA array findings, qRT-PCR demonstrated that *circPtchd2* expression was indeed significantly higher in SAC female vs. SAC male brains and that this sex-dimorphic expression was lost following PAE (Figure 4C). However, qRT-PCR did not find significant increases in male PAE vs. SAC as shown in the circRNA array data (Figure 4C and Supplementary Table 1), verifying the semi-quantitative nature of circRNA profiling. As a positive control, we also measured the female-specific lncRNA Xist, which was only expressed in female brains in our samples regardless of treatment (Figure 4D). In order to determine if changes in these circRNAs are accompanied by similar changes in mRNA expression from the same genes, we performed reverse transcription with oligo-dt primers to enrich for poly-A tail mRNAs and used qRT-PCR to measure the overall levels of *Ptchd2* and *Satb2* mRNAs in the same E18 brain samples. We found that there were no changes in the expression of both of these mRNAs (Figures 4E,F). Moreover, there was no significant correlation between the expression *circSatb2* and *Satb2* mRNA (*r* = + 0.2982, *p* = 0.2293, two-tailed Spearman’s correlation) and a trend of a positive association between *circPtchd2* and *Ptchd2* mRNA (*r* = + 0.4140, *p* = 0.0780, two-tailed Spearman’s correlation). Taken together these data suggest that sex-specific circRNA expression in prenatal brain and effects on circRNA levels by PAE are not associated with changes in mRNA expression.

### Changes in *CircPtchd2* and *CircSatb2* Expression Display Differential Correlations With Neuronal Marker Expression

**FIGURE 5:**
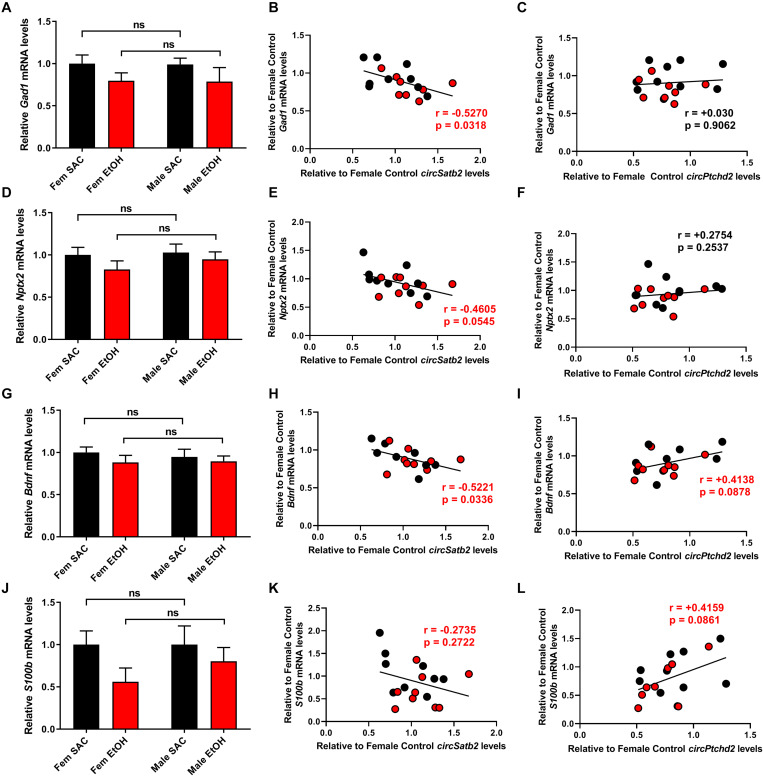
Changes in *circPtchd2* and *circSatb2* expression in prenatal brain are correlated with neuronal and glial gene expression. **(A,D,G,J)** Bar graphs showing mean + SEM of neuronal and glial gene mRNAs relative to female SAC (control), based on qRT-PCR and normalized to *18SrRNA*. No sex-specific significant difference between control (black bars) and PAE groups (red bars) was observed in **(A)** *Gad1*, **(D)** *Nptx2*, **(G)** *Bdnf*, and **(J)** S100b mRNA levels. **(B–L)** Correlations (two-tailed Spearman’s) between *circSatb2* and *Gad1*, *Nptx2*, *Bdnf*, and *S100b* mRNA expression **(B,E,H,K)** and *circPtcd2* and *Gad1*, *Nptx2*, *Bdnf*, and *S100b* mRNA expression **(C,F,I,L)** mRNA levels. For **(A,D,G,J)**, *N* = 5–6 per group. Male SAC, female SAC, male PAE, and female PAE samples are all included in the correlations shown.

*Ptchd2* is a brain-enriched gene regulated by the thyroid hormone that has been shown to be essential for the self-renewing capacity of neuronal stem cells and whose knockdown precautiously increases both neuronal and glial differentiation (Konířová et al., 2017). Similarly, *Satb2* is a gene important for callosal upper-layer neuron specification that is also implicated in FASD (Alcamo et al., 2008; Britanova et al., 2008; Rosenfeld et al., 2009; Mandal et al., 2015). Given the above, we measured the expression of several neuronal and glial markers via qRT-PCR and correlated their levels with *circPtchd2* and *circSatb2* expression in male and female SAC and PAE E18 whole brains (Figures 5A–L). Interestingly, we found a significant or near significant inverse correlation between *circSatb2*, but not *circPtchd2*, and excitatory and inhibitory neuronal gene expression as measured via Neuronal pentraxin-2 (*Nptx2*) and Glutamate decarboxylase 1 (*Gad1*) mRNA levels (Figures 5B,C,E,F). Furthermore, changes in S100 calcium-binding protein b (*S100b*), a gene enriched in astrocytes and radial glia, and brain-derived neurotrophic factor (*bdnf*; expressed in both neurons and glia) mRNA expression showed a trend for positive correlation with *circPtchd2*, while *circSatb2* exhibited a negative association with *Bdnf* levels in E18 whole brain (Figures 5H,I,K,L). However, none of the measured mRNAs displayed any significant changes by either sex or PAE (Figures 5A,D,G,J). Taken together, our data suggest that *circPtchd2* and *circSatb2* display differential correlations with neuronal gene expression.

## Conclusion

Emerging studies suggest that circRNAs are abundantly expressed in the brain and could be important regulators of neuronal development and function. However, the role of circRNAs in neurodevelopmental disorders remains poorly understood. Here, we performed circRNA profiling and RNA seq in E18 whole brain of male and female mice subjected to PAE. Our results uncovered sex-specific changes in whole brain circRNA but not mRNA expression as a result of PAE, and demonstrated an overall trend for PAE to erase or reverse the sex dimorphic expression of the majority of circRNAs expressed at E18 whole brains. Moreover, we show that differentially expressed by PAE circRNAs were preferentially derived from genes with important roles in brain development and function, with male PAE-altered circRNAs being more likely to be generated from genes involved in later stages of neuronal development than female PAE-altered circRNAs. In addition, we validated the sex specific changes in *circSatb2* as a result of PAE and demonstrated the sex dimorphic expression of *circPtchd2* in control E18 brains, which was lost following PAE treatment.

A subset of previous studies have shown sex-specific effects of PAE in the placenta and developing brain (Weinberg et al., 2008; Loke et al., 2018) and a potential sex dichotomy in PAE-induced alterations in the hypothalamic-pituitary-adrenal (HPA) axis (Terasaki et al., 2016). However, the majority of animal models of FASD have only focused in male mice. Our study, is the first thus far to uncover a sex-specific effect in circRNA but not mRNA expression in the developing brain as a result of PAE. Given that circRNA synthesis can be regulated independently of protein-coding gene expression, it is possible that PAE can result in alterations in circRNA biogenesis factors in a sex-specific manner. Future studies are needed however, to examine the exact molecular mechanisms that could underlie such an effect. Furthermore, our data suggested that the majority of PAE-mediated changes in circRNA but not mRNA expression in either male of female E18 whole brains tended to either normalize or reverse sex-specific changes observed in control brains. This unexpected finding suggests that circRNAs could be particularly sensitive to the sex dimorphic effects of PAE in the developing brain. Additional research is needed, however, to determine whether changes in circRNA expression are important mediators of sex-specific changes in neuronal development and function in FASD.

Our findings of increased expression of *circPtchd2* in control female vs. male E18 brains and a lack of sex-specificity in the levels of this circRNA following PAE are of interest, since *Ptchd2* is a gene with known importance for early brain development (Konířová et al., 2017). Furthermore, *Ptchd2* (also known as *Disp3*) is a sterol-sensing domain-containing protein that is regulated by the thyroid hormone (Zikova et al., 2009). Given that previous data have suggested that prenatal thyroid hormone treatment can reverse some of the behavioral abnormalities observed following PAE (Wilcoxon et al., 2005), it is tempting to hypothesize that *circPtchd2* synthesis could also be affected by thyroid hormone signaling. In addition, the observed male-specific increases in *circSatb2* expression after PAE are of particular importance, given the relevance of the parent gene (S*atb2*) in brain development and FASD (Alcamo et al., 2008; Britanova et al., 2008; Rosenfeld et al., 2009; Mandal et al., 2015). Although, we have yet not elucidated the exact mechanisms that could specifically alter circRNAs in a sex-specific manner, it is worth noting that ADAR1, one of the major inhibitors of neuronal circRNA biogenesis (Rybak-Wolf et al., 2014) is affected by androgen receptor signaling (Shi et al., 2017). Moreover, quaking (QKI) is a well-established positive regulator of circRNA biogenesis (Conn et al., 2015) that is also shown to be affected by alcohol consumption in the liver (Zhang et al., 2019). It is, therefore, possible that different circRNA biogenesis genes could be regulated by sex and exposure to alcohol to generate sex-dimorphic alterations in circRNA expression in the developing brain.

The observed inverse correlations between *circSatb2* and excitatory and inhibitory neuronal gene expression and the differential associations of *circPtchd2* and *circSatb2* with *Bdnf* expression, suggest the possibility that these circRNAs could be involved in sex-specific molecular pathways regulating neurogenesis and radial glial function in the developing brain. We have previously shown that brain enriched circRNAs related to psychiatric disorders could regulate neuronal gene expression independently of their linear mRNA counterparts (Zimmerman et al., 2020). It is, therefore, worth investigating whether these two circRNAs can have additional effects in brain development and function other than what is expected by the known function of the *Ptchd2* and *Satb2* genes. However, it is also possible that such correlations are a result of different cell-specific expression of these two circRNAs. Future studies utilizing *in vivo* circRNA-specific manipulations of these circRNAs combined with detailed analysis of brain development and behavior are needed to dissect the relevance of such circRNAs for sex-dimorphism in brain development and for the sex-specific effects of PAE.

Taken together, our study demonstrates that circRNAs are regulated in a sex-specific manner in the developing brain and are differentially affected by prenatal exposure to alcohol. Combined our work provides the first evidence of alterations in circRNA expression in the brain as a result of PAE and introduces novel molecular players with potential importance to FASD.

## Data Availability Statement

The datasets presented in this study can be found in online repositories. The names of the repository/repositories and accession number(s) can be found below: https://www.ncbi.nlm.nih.gov/ (GSE154018); https://data.mendeley.com/datasets/yff4ggkd2y/1.

## Ethics Statement

All procedures were approved by the University of New Mexico Health Sciences Center Institutional Animal Care and Use Committee.

## Author Contributions

NM conceived the hypothesis, designed and supervised experiments, analyzed the data, and wrote the manuscript. AA, KC, and AG designed and executed experiments and provided feedback on the manuscript. PP designed and executed experiments, analyzed data and co-wrote the manuscript. SA, EL, and CP designed and executed experiments and analyzed data. All authors contributed to the article and approved the submitted version.

## Conflict of Interest

The authors declare that the research was conducted in the absence of any commercial or financial relationships that could be construed as a potential conflict of interest. The reviewer RM declared a past co-authorship with one of the authors AA to the handling editor.

## Footnotes

1 https://data.mendeley.com/datasets/yff4ggkd2y/1
